# The impossibility of reliably determining the authenticity of desires: implications for informed consent

**DOI:** 10.1007/s11019-017-9783-0

**Published:** 2017-06-08

**Authors:** Jesper Ahlin

**Affiliations:** Division of Philosophy, KTH Royal Institute of Technology, Brinellvägen 32, 100 44 Stockholm, Sweden

**Keywords:** Authenticity, Autonomy, Informed consent, Decision-making, Healthcare

## Abstract

It is sometimes argued that autonomous decision-making requires that the decision-maker’s desires are authentic, i.e., “genuine,” “truly her own,” “not out of character,” or similar. In this article, it is argued that a method to reliably determine the authenticity (or inauthenticity) of a desire cannot be developed. A taxonomy of characteristics displayed by different theories of authenticity is introduced and applied to evaluate such theories categorically, in contrast to the prior approach of treating them individually. The conclusion is drawn that, in practice, the authenticity of desires cannot be reliably determined. It is suggested that authenticity should therefore not be employed in informed consent practices in healthcare.

## Introduction

Informed consent is a patient’s valid authorization or refusal of a medical intervention; a process aiming at protecting patients’ autonomy. In its elaborate form it is usually understood as informed, voluntary, and competent consent (cf. Eyal 2012). Clinicians sometimes meet patients who are competent, yet make (at least seemingly) incomprehensible treatment decisions.^1^ Some of those decisions can be described as *inauthentic*.

The question can be raised whether the authenticity of decisions should be included as a criterion in informed consent to further protect patients with regards to their autonomy.^2^ In this article, I argue that the authenticity (or inauthenticity) of desires cannot be reliably detected. Therefore, authenticity should not be part of informed consent. A well-founded suspicion that a desire is inauthentic may call for other measures than the invalidation of consent (or refusal), such as a moral obligation to double-check that the patient is competent to make healthcare decisions. However, the aim of this article is mainly theoretical. Although some possible policy implications are suggested, none is defended at length.

The paper is structured as follows. In “The problem of authenticity and informed consent”, I elaborate on the problem of authenticity and informed consent. In “A taxonomy of authenticity”, I introduce a taxonomy of characteristics displayed by theories of authenticity. In “The taxonomy and the argument from testability”, I use the taxonomy to evaluate the prospect of different theories of authenticity to produce reliably observable consequences. Lastly, "Concluding remarks” contains some concluding remarks.

## The problem of authenticity and informed consent

### Anna

Consider the hypothetical case of Anna, a young and promising professional ballet dancer. Anna loves her work. She has moved across the nation to attend the best ballet schools, set aside personal relationships when they conflict with her career, and is known by friends and family to love dancing “more than anything else.” Anna has suffered a serious leg injury. To avoid the risk of having to go through an amputation that will definitely end her career as a dancer, she must undergo a minor surgery. She understands information relevant to her condition, is capable to reason about the potential risks and benefits of different treatment alternatives, appreciates the nature of her situation, the consequences of her choices, and so on. Yet, she refuses to undergo surgery.

There is no physiological or psychological disorder, such as a brain tumor, untreated syphilis, or psychosis, that can be tied to Anna’s decision-making. Neither is she being forced or unduly influenced to make a decision that accords with someone else’s interests, certain social relations, authoritative traditions, or anything else that might impinge on the voluntariness of her choices. She plainly refuses to undergo surgery.

When reflecting upon the case, her doctor considers Anna’s treatment decisions to be “out of character.” She believes that Anna is not being “herself,” which is why she makes choices that are not “genuine.” Nonetheless, the doctor must conclude, Anna is competent to make treatment decisions; nothing in the informed consent process would allow anyone to override Anna’s choices. However, if informed consent had included a criterion of *authenticity*, Anna’s decisions could have been invalidated on that basis. Her “true wishes” could then be adhered to by forcing the measures necessary to save Anna from amputation. Therefore, the doctor contemplates whether or not the authenticity of patients’ decisions should be part of informed consent.

The question arises in various contexts. For instance, anorexia nervosa patients have stated that the disorder “was a part of themselves, and therefore it would not be them if they recovered” (Tan et al. 2006b, p. 278). Similarly, some people with bipolar disorder have been reported to ask whether certain experiences are due to their illness, medication, or themselves (Hope et al. 2011, p. 21). And, brain tumors can entail personality changes, such as in the case of a 40-year-old male who suddenly developed pedophilia (Burns and Swerdlow 2003). All examples of cases in which the concept of authenticity can be invoked.

### Authentic desires and informed consent

There are several interrelated problems concerning the question of whether the authenticity of patients’ decisions should be part of informed consent. First, it must be determined what authenticity is. Lexical definitions of “authentic” include descriptions such as “real or genuine,” “not copied or false,” “true and accurate,” and so on, but for moral reasons it is necessary to adopt a more detailed and systematized account, i.e., a *theory* of authenticity.^3^ Second, a method must be developed that enables observers to reliably recognize authenticity (or inauthenticity) in others. Merely having a theory of authenticity does not suffice, as the concept is (or is not) to be applied in a context in which interpersonal morality requires that interventions with other people’s lives and liberties are justified. It is first when these two matters are satisfactorily settled that we are in a position to judge whether or not to include authenticity in informed consent.

This article treats the second of the above stated problems. Thus, I do not aim to contribute to the philosophy of authenticity—although I believe that this work does so indirectly—but merely to its applicability. I claim to show that a method that enables observers to reliably recognize authenticity (or inauthenticity) in others cannot be developed. However, this claim must be conditioned. First, I only take into consideration theories of authenticity present in contemporary literature on personal autonomy. Second, my claim is delimited by the fact that I only treat theories in what is commonly called a *procedural* tradition of personal autonomy, which can be contrasted with a *substantial* tradition. In the procedural tradition, theorists are only concerned with the process by which desires are formed and realized. In the substantial tradition, theorists are also concerned with the content of a desire-holder’s desires (see e.g. Oshana 2015). Third, I assume that authentic desires can be treated without a well-articulated idea of what it is to be an authentic *person*. This assumption requires some elaboration.

Much of what has been said of authenticity is phrased as “preferences stemming from her *true self*,” and similar. The problem with such phrases is that they necessitate some idea of personhood. In the humanities, it is a frequently debated problem what personhood is, or what it is to be a person. Are we socially constituted beings, as some believe, or are we self-made? Is *tabula rasa* a real thing? And, in all cases, to what extent? I think that the current problem is possible to treat without engaging in such debates. That is, it should not matter to my argument or to informed consent whether humans are socially constructed beings or if we are something else. Whatever we are, I am here concerned only with *desires*. In this context, I intend for desires to be understood as the basic element in preference forming, i.e., basic pro-attitudes. Therefore, I treat theories of authenticity as theories of authentic desires—although these often include a mix of propositions about “authentic selves,” “authentic decisions,” “authentic preferences,” and so on.

### Method

I approach the problem as follows. Sjöstrand and Juth recently concluded that the concept of authenticity is “highly problematic to use as a criterion for autonomous decision-making in healthcare” (2014, p. 115). Although I agree with them, it is not my intention to merely reproduce their arguments here. I wish to strengthen their conclusion with new arguments. Sjöstrand and Juth only treat authenticity in the context of psychiatric care. However, I use a method that allows me to conclude that authenticity is problematic in the above sense in all healthcare settings. My method requires a more in-depth explanation of the problem at hand.

Sjöstrand and Juth write the following (p. 121):

The practical question is which patients should be deemed inauthentic enough not to be granted certain rights typically granted to patients considered fully autonomous—for instance, a right to refuse treatment. Hence, we also need to have some idea about how to *test* patients regarding the authenticity of their desires. This seems to be very difficult…

I call this *the argument from testability*. It is further developed in “The argument from testability”. Here, it suffices to declare that it is more significant than Sjöstrand and Juth acknowledges. First, testability is central to the problem of developing a method that enables observers to reliably recognize authenticity (or inauthenticity) in others. Second, the argument from testability applies in some form not only to the theory of authenticity favored by Sjöstrand and Juth. If my thesis holds, the argument from testability applies universally, and authenticity cannot be reliably employed as a criterion in informed consent practices.

As stated above, I use a different method than Sjöstrand and Juth’s. They go through a collection of theories of authenticity individually and demonstrate in each case how that specific theory is flawed. One problem with that method is that it is space consuming. It requires of the authors to briefly summarize each theory—which paves the way for misrepresentations—and to, just as briefly, demonstrate precisely what is wrong with it. Another problem is that many theories may be left out of the analysis. By contrast, in this article, I introduce a taxonomy of characteristics displayed by different theories of authenticity that allows me to treat such theories categorically. The method is less space consuming and its results more reliable, although it cannot be guaranteed that the taxonomy covers all conceivable characteristics of authenticity. Nonetheless, my method collects many theories of authenticity, several of which have been highly influential, and makes their similarities and differences comprehensible.^4^ Even if my conclusion is unconvincing, the taxonomy is still a valuable contribution to the discussion of authenticity in autonomy theory.

## A taxonomy of authenticity

### The taxonomy

There are many theories of authenticity.^5^ As is made clear above, I will not attempt to go through them all here. However, I will account for some distinctive elements that many theories share. This allows me to organize characteristics displayed by different theories of authenticity into tree distinct categories: *sanctionist, originist*, and *coherentist*. These are not formal definitions, but broad categories that distinguish different conceptualizations of authenticity. In sanctionist theories, i.e., theories distinguished by characteristics typical of sanctionist ideals, authenticity concerns the desire-holder’s attitude towards her desires. In originist theories, authenticity concerns the origin of a desire. In coherentist theories, authenticity concerns the coherence of a desire-holder’s set of desires. This will be elaborated below. Furthermore, these categories come in two classes: *cognitivist* and *non-cognitivist*. In cognitivist theories, authenticity is a matter of rational deliberation; non-cognitivist theories do not commit to that. Thereby, non-cognitivist theories do not reject rational deliberation altogether, they merely do not commit to the narrow view that authenticity is only a matter of rational deliberation. A theory of authenticity can display characteristics from more than one category. The classes on the other hand are mutually exclusive, so that a theory is either one or the other.

Thereby, the taxonomy takes the form of a three-by-two scheme.^6^ I will go through the different categories and classes respectively, and illustrate their distinct features by using quotes and examples from theories that display elements that are characteristic for each category and class.

### Sanctionism

In sanctionist theories, authenticity concerns the desire-holder’s attitude towards her desires. Desires that in one way or another are sanctioned by the desire-holder are deemed authentic. Consider, for instance, Frankfurt, whose idea of a *person* is that such a being identifies reflectively with her desires, and Dworkin, who holds that it is crucial to a person’s autonomy that she has the “capacity to raise the question of whether [she] will identify with or reject the reasons for which [she] now act[s]” (Frankfurt 1971, pp. 10–17; Dworkin 1988, p. 15). Similarly, Juth writes that “the most important property of an authentic desire is that a person who has the desire would be inclined to approve of having that desire if she came to know why she has it” (2005, p. 129). This is also the type of theory that Sjöstrand and Juth favors: it is “the person’s own attitude towards the desire in the light of knowledge about the origin that matters” (2014, p. 121).

According to sanctionist theories, the status of a desire in terms of authenticity is determined by means of endorsement. Suppose that Anna came to know exactly why she has the desire to refuse to undergo the minor surgery that is necessary to avoid the risk of amputation. In this hypothetical state of mind, she is aware of everything that might subconsciously influence her desire forming; nothing regarding her psychological and physiological behavioral patterns escapes her internal gaze. Sanctionist theories suggest that Anna’s desires are authentic if and only if Anna, in this hypothetical state of mind, would endorse the reasons for why she has the desire in question.

The above are examples of *cognitivist* sanctionist theories of authenticity. According to them, authenticity is a matter of rational deliberation. Frankfurt suggests that persons identify *reflectively* with their desires and Dworkin writes about a “capacity to *raise the question*” (emphasis added; see quote above). Accordingly, Sjöstrand and Juth use the label “*Rationally* endorsed desires” to describe theories such as these (p. 120; emphasis added). I know of no *non-cognitivist* sanctionist theories, but the taxonomy may allow us to formulate one. A theory could, perhaps, be developed so that a desire is authentic only if the desire-holder experiences an emotional inclination in favor of it.

### Originism

In originist theories, authenticity concerns the origin of a desire. In a manuscript, Tan et al. formulate an originist theory of authenticity as a counterfactual statement: Authentic views are such that a person “would have (or did have) if she did not suffer from [a disorder]” (2006a, p. 20).^7^ Similarly, but more elaborately, Elster argues when writing about the rationality of desires that desires are inauthentic if they are “shaped by irrelevant causal factors, by a blind psychic causality operating ‘behind the back’ of the person” (1983, p. 16; Sjöstrand and Juth 2014, p. 118). All desires have a “causal origin, but some of them have the wrong sort of causal history” (Elster 1983, p. 16). Elster continues by writing about persons that “are in control over the processes whereby their desires are formed,” stating that “autonomous [here: authentic] desires are desires that have been deliberately chosen, acquired or modified—either by an act of will or by a process of character planning” (p. 21). Thus, according to Elster, authentic desires are such that originate in some cognitive process controlled by the desire-holder. That is, Anna’s desire to refuse to undergo surgery to avoid the risk of amputation could originate in something that is beyond her cognitive control.

An example of an originist theory of authenticity that can be interpreted as non-cognitivist is found in Meyers. Arguing against Frankfurt (see above), Meyers writes that having “an authentic self is best understood as the result of an ongoing activity of persons” (2001, p. 199). The authentic self is “the evolving collocation of attributes—analogous to a musical ensemble’s sound—that issues from ongoing exercise of” a repertory of skills of “introspection, imagination, memory, communication, reasoning, interpretation, and volition” that enable self-discovery and self-definition (ibid). Elsewhere, Meyers writes that when exercising such skills one “*enacts* one’s authentic self” (2005, p. 49). Although the theory is built on a cognitivist foundation, it is ultimately non-cognitivist. Meyers writes that what “autonomous people do to understand and define themselves is not aptly figured by any Euclidean shape or formal reasoning pattern” (2001, p. 199). Thus, enacting one’s authentic self is not a rationalist enterprise. A Meyerean theory of authenticity phrased in terms of desires could be formulated accordingly: desires are authentic if and only if they originate in non-cognitivist processes of self-discovery and self-definition.

### Coherentism

In coherentist theories, authenticity concerns the coherence of a desire-holder’s set of desires. Christman argues that for a characteristic to be authentic it must pass a self-critical reflection, similar to that in cognitivist sanctionist theories. However, the reflection does here not target the rational endorsement of having a certain desire, but whether the characteristic in question can be “sustained as part of an acceptable autobiographical narrative organized by her diachronic practical identity” (2009, p. 155). While sanctionism is an atomist theory focusing on individual desires, coherentism is holist; authenticity here concerns a whole body of desires.

Phrased in terms of desires, a Christmanean theory of authenticity could be that a person’s desires are authentic if and only if they fit with her socio-historical or autobiographical narrative. Anna’s desire to refuse to undergo surgery does not fit with her socio-historical or autobiographical narrative. She loves do dance “more than anything else,” is known to have set aside personal relationships when they have conflicted with her career, and so on. Her present desires just do not *fit*.

The Christmanean theory is cognitivist. Similarly, albeit as an example of a non-cognitivist coherentist theory, Miller writes (1981, p. 24):

Autonomy as authenticity means that an action is consistent with the person’s attitudes, values, dispositions, and life plans. Roughly, the person is acting in character. … For an action to be labeled”inauthentic” it has to be unusual or unexpected, relatively important in itself or its consequences, and have no apparent or proffered explanation.

These are the categories and classes of characteristics displayed by different theories of authenticity. Below, the taxonomy is used to test such theories categorically.

## The taxonomy and the argument from testability

### The argument from testability

Most propositions and theories can be tested in several ways. One test could, for instance, aim at identifying conceptual vaguenesses, ambiguities, and inconsistencies in theories of authenticity. The concern of the argument from testability, however, is something else. Theories of authenticity will here not be evaluated as such. Since authenticity is (or is not) to be applied in informed consent contexts, it is a necessary criterion of a theory of authenticity that it renders observable and testable consequences. Therefore, it is only the prospect of the theories producing empirically observable consequences, and the possibility of evaluating those consequences, that is of interest here. Contemporary theories of authenticity may be good in other respects, although it is beyond the present purpose to assess that.

The taxonomy of characteristics displayed by different theories of authenticity allows us to evaluate the testability of theories of authenticity categorically. If it is true that neither sanctionist, originist, nor coherentist characteristics can produce observable and testable consequences, no theory that builds on those elements and those elements only achieves the requirement posed by the argument from testability. In “Sanctionism” through “Coherentism”, I spell out what the argument from testability requires of each category of characteristics, and show that no such category passes the test.

### Sanctionism

Suppose that Anna’s doctor is a sanctionist regarding authenticity. She believes that for a desire to be authentic it must be hypothetically endorsed by the desire-holder. There are two main reasons why this view does not render any observable and testable consequences. First, as Sjöstrand and Juth write (p. 121):

For one thing, it is often difficult to come up with a full explanation as to why we have a certain desire, and even more difficult to make the necessary investigations in order to determine whether or not this explanation is correct.

This practical problem may be overcome, as discussed in “Originism” below. But, in sanctionist theories, desire-holders are to transcend into a state of mind from which the status of a desire is assessed. There are two possibilities here. Either that state of mind is hypothetical, in which case the theory cannot render observable consequences (but merely hypothetical ones). Sanctionist theories are then not falsifiable. Or, it is an actual state of mind. If it is an actual state of mind, observers must evaluate whether the desire-holder transcends into *it*, into some *other* state of mind, or if she does not transcend into anything at all. Furthermore, they must reliably determine whether valid endorsement is actually taking place when the desire-holder is in that state of mind. To do so would require access to advanced (and currently unavailable) neuro-imaging technology, in addition to an in-depth knowledge of the psychological nature of endorsement. It would appear that sanctionism is, at the very least, impractical.

In conclusion, sanctionism does not render observable and testable consequences without technology and scientific knowledge yet unheard of, if at all. That entails that, at least as of today, a method that enables observers to reliably recognize authenticity (or inauthenticity) in others cannot be grounded in sanctionist theories of authenticity only.

### Originism

Suppose instead that Anna’s doctor is an originist regarding authenticity. She believes that for a desire to be authentic it must originate in a process controlled by the desire-holder. In practice, this view also fails to render observable and testable consequences.^8^

Again quoting Sjöstrand and Juth, it is difficult “to come up with a full explanation as to why we have a certain desire, and even more difficult to make the necessary investigations in order to determine whether or not this explanation is correct.” Observers face the insurmountable task of tracing the origin of desires in hindsight and attempt to reliably determine when they were formed. And, if that problem is resolved and the time of origin detected, observers must also reliably determine whether the desire-holder was in control over the desire-forming process at the time.

These problems are significant in theory, but plausibly impossible to overcome in practice. Against scarcity of resources, healthcare practices would have to develop manageable and effective methods to examine the origin of desires. Among other things, those methods would likely have to include deep psychological analysis and a detailed socio-historical biographical investigation. In addition to that, to determine whether the desire-holder was in control of the desire-forming process, it is likely the case that the methods would have to include interviews with people who were close to the desire-holder when the desires were initially formed, and other similar measures. To complicate things further, these investigations would also require the desire-holder’s informed consent.

To conclude, originist theories may render observable and testable consequences in theory. However, to examine the matter would require overwhelmingly complex and resource-demanding methods. Therefore, it is plausibly insurmountably difficult for healthcare practices to reliably recognize originist authenticity (or inauthenticity) in patients.

### Coherentism

Suppose, then, that Anna’s doctor is a coherentist regarding authenticity. She believes that authenticity concerns the coherence of a desire-holder’s set of desires. Naturally, she thinks of Anna’s desire to refuse to undergo minor surgery to avoid the risk of amputation as diverging. In short, the desire does not fit.

Assessing the authenticity of Anna’s desire requires an exhaustive list of her desires. In addition to her desire to move across the country, attend the best ballet schools, and set aside personal relationships that conflict with her career, it must include desires that may arise in situations not immediately or obviously connected to the present one. The set must also include desires in unknown situations, e.g., such that will arise in the future and of which nothing can be known. It cannot be determined when a desire-set is full. Therefore, observers cannot reliably determine the coherence of a specific desire.

*Prima facie*, a reasonable way to circumvent the problem of composing an exhaustive desire-set is to in some way delimit the extent of the set, although a reflected judgment reveals that doing so implies making normatively substantial decisions. Delimiting the set necessitates deciding that some desires are irrelevant to the assessment. In fact, coherentism is inherently normative (cf. Banner and Szmukler 2013, p. 390). It cannot be explained why a diverging desire is inauthentic rather than the rest of the desire-holder’s set of desires, without invoking the support of normative auxiliary assumptions. That is, Anna’s doctor cannot be sure that it is not Anna’s desires to move across the country, attend the best ballet schools, and set aside personal relationships that conflict with her career that are inauthentic. Empirical data, or incoherency as such, do not reveal which piece of the desire pie that should be discarded; the large or the small one. The truth of the matter cannot be *discovered*, but must be *decided*.

An intuitively compelling example that corresponds to the case of Anna is a person who suddenly reveals that she is homosexual, to the surprise of everyone close to her. Her romantic desire toward others of the same sex cannot be thought of as “inauthentic” only because it deviates from her previously displayed desires, unless some normative auxiliary assumption is invoked in favor of the largest piece of the desire pie. Therefore, coherentism is an inherently normative characteristic in authenticity theory.

In conclusion, even if the problem of composing an exhaustive desire-set is overcome, coherentist characteristics do not render observable and testable consequences independent from normative auxiliary assumptions. Therefore, a method that enables observers to reliably recognize authenticity (or inauthenticity) in others cannot be grounded in coherentist theories of authenticity only; it also requires a moral defense.

## Concluding remarks

Above, it has been shown that theories that build on characteristics covered by the taxonomy fail to meet the requirements set by the argument from testability. However, that does not imply that we can be sure that authenticity cannot be part of informed consent. There might be characteristics and theories that the taxonomy here introduced does not cover. Furthermore, my assumption that authentic desires can be analyzed without a well-articulated idea of authentic persons may be mistaken. The same applies to my choice to only treat theories of authenticity in the procedural tradition of personal autonomy theory. Substantial theories of authenticity have been left out of the present analysis; they may succeed where procedural theories do not. Lastly, the alternative remains to begin with what can be reliably detected regarding desires and develop a theory of authenticity thereafter—that is, to intentionally put the cart before the horse.

However, if my assumptions are sound and the taxonomy is exhaustive, in practice, the authenticity (or inauthenticity) of desires cannot be reliably detected. Therefore, authenticity should not be included as a criterion in informed consent.

Nonetheless, seemingly inauthentic behavior from patients may trigger the need to take other actions than invalidating consent (or refusal). Anna’s doctor may, for instance, be morally obliged to double-check that Anna is able to comprehend the nature of her situation. Or, surprising desires such as Anna’s might prompt the need for alternative communicative measures, e.g., the use of pedagogical tools, or perhaps another doctor’s affirmation that the information the patient has received is correct. However, it is beyond the limits of this article to further treat moral obligations that may arise from a suspicion of inauthenticity. Any detailed policy suggestions based on the conclusions drawn in this article must be carefully but separately formulated.

## Footnotes

1 Competent according to e.g. the MacCAT-T: a clinical tool to assess patients’ capacities to make treatment decisions. See Grisso et al. (1997).

2 See e.g. O’Shea, who raises the possibility of introducing authenticity as a necessary condition of consent in order to distinguish between benign persuasion and undue influence (2011, pp. 30–31).

3 These descriptions are from Merriam-Webster online. The arguments in this article do not commit to any specific lexical definition of “authenticity,” but treats a number of suggestions that have been proposed with regards to how the concept should be understood.

4 I am not aware of any theory that the taxonomy does not cover.

5 In addition to those explicitly mentioned in this article, see e.g. Buchanan and Brock (1990), Chariand (2001), DeGrazia (2005), Faden and Beauchamp (1986), Freedman (1981), Tännsjö (1999), Velleman (2002), Winnicott (2007).

6 A third dimension could be added to the taxonomy, marking the degree to which a theory displays the characteristic in question. However, my argument does not require such elaborations and it will therefore be left out of the analysis.

7 This is omitted in the published version of the article (Tan et al. 2006b).

8 As pointed out by an anonymous reviewer, the question of tracing the origins of a desire may, at least partially, be metaphysical rather than (socio-)psychological. That may be true, but metaphysical theses are not empirically testable, so I choose here not to address the possibility of metaphysical origins of desires.

## References

[CR1] Banner NF, Szmukler G (2013). ‘Radical Interpretation’ and the assessment of decision-making capacity. Journal of Applied Philosophy.

[CR2] Buchanan AE, Brock DW (1990). Deciding for others: The ethics of surrogate decision making.

[CR3] Burns JM, Swerdlow RH (2003). Right orbitofrontal tumor with pedophilia symptom and constructional apraxia sign. Archives of Neurology.

[CR4] Charland LC (2001). Mental competence and value: The problem of normativity in the assessment of decision-making capacity. Psychiatry, Psychology and Law.

[CR5] Christman J (2009). The politics of persons: Individual autonomy and socio-historical selves.

[CR6] DeGrazia D (2005). Human identity and bioethics.

[CR7] Dworkin G (1988). The theory and practice of autonomy.

[CR8] Elster J (1983). Sour grapes: Studies in the subversion of rationality.

[CR9] Eyal, N. 2012. Informed Consent. *Stanford Encyclopedia of Philosophy*. Retrieved from http://plato.stanford.edu/entries/informed-consent/.

[CR10] Faden R, Beauchamp T (1986). A History and theory of informed consent.

[CR11] Frankfurt H (1971). Freedom of the Will and the Concept of a Person. The Journal of Philosophy.

[CR12] Freedman B (1981). Competence, marginal and otherwise: Concepts and ethics. International Journal of Law and Psychiatry.

[CR13] Grisso T, Appelbaum PS, Hill-Fotouhi C (1997). The MacCAT-T: A clinical tool to assess patients’ capacities to make treatment decisions. Psychiatric Services.

[CR14] Hope PT, Tan DJOA, Stewart DA, Fitzpatrick PR (2011). Anorexia nervosa and the language of authenticity. Hastings Center Report.

[CR15] Juth, N. 2005. *Genetic Information—Values and Rights. The Morality of Presymptomatic Genetic Testing*: Acta Universitatis Gothoburgensis.

[CR16] Meyers DT, Elevitch B (2001). Authenticity for real people. Proceedings of the twentieth world congress of philosophy: Philosophy of mind and philosophy of psychology.

[CR17] Meyers DT, Christman J, Anderson J (2005). Decentralizing Autonomy: Five Faces of Selfhood. Autonomy and the challenges to liberalism.

[CR18] Miller BL (1981). Autonomy and the refusal of lifesaving treatment. The Hastings Center Report.

[CR19] O’Shea, T. 2011. Consent in History, Theory and Practice *Essex Autonomy Project Green Paper Report*: University of Essex: Essex Autonomy Project.

[CR20] Oshana MAL, Oshana MAL (2015). Is social-relational autonomy a plausible ideal?. Personal autonomy and social oppression: Philosophical perspectives.

[CR21] Sjöstrand M, Juth N (2014). Authenticity and psychiatric disorder: does autonomy of personal preferences matter?. Medicine, Health Care and Philosophy.

[CR22] Tan, D. J. O. A., Hope, P. T., Stewart, D. A., and Fitzpatrick, P. R. 2006a. Competence to make treatment decisions in anorexia nervosa: thinking processes and values. Retrieved from https://www.ncbi.nlm.nih.gov/pmc/articles/PMC2121578/pdf/nihms5623.pdf. 10.1353/ppp.2007.0032 PMC2121578 18066393

[CR23] Tan DJOA, Hope PT, Stewart DA, Fitzpatrick PR (2006). Competence to make treatment decisions in anorexia nervosa: thinking processes and values. Philosophy, Psychiatry, and Psychology: PPP.

[CR24] Tännsjö T (1999). Coercive care: The ethics of choice in health and medicine.

[CR25] Velleman DJ, Buss S, Overton L (2002). Identification and Identity. Contours of agency: Essays on themes from Harry Frankfurt.

[CR26] Winnicott DW (2007). Ego distortion in terms of true and false self *the maturational process and the facilitating environment: Studies in the theory of emotional development*.

